# Identification and pathogenicity of hepatitis E Virus from laboratory Bama miniature pigs

**DOI:** 10.1186/s12917-022-03206-7

**Published:** 2022-03-15

**Authors:** Baoyuan Liu, Yiyang Chen, Meimei Zhang, Tianxiang Chen, Yuan Zhang, DanBaZhaXi, Shixuan Xu, Qin Zhao, En-Min Zhou

**Affiliations:** 1 Department of Preventive Veterinary Medicine, College of Veterinary Medicine, Northwest A&F University, Yangling, Shaanxi China; 2 Scientific Observing and Experimental Station of Veterinary Pharmacology and Diagnostic Technology, Ministry of Agriculture, Yangling, Shaanxi China; 3 General Station of Animal Husbandry and Veterinary Technology Promotion, Naqu, Tibet China

**Keywords:** Hepatitis E virus, Bama miniature pig, Sequence analysis, Pathogenicity

## Abstract

**Background:**

Hepatitis E virus (HEV) genotypes 3 and 4 are zoonotic. In this study, HEV infection in laboratory Bama miniature pigs in Sichuan Province of China was investigated. Firstly, one hundred rectal swabs were collected for HEV RNA testing, and chose positive samples for sequence analysis. Concurrently, for pathogenicity study, six healthy Bama miniature pigs were randomly divided into two groups of 3 pigs each. A total of 500 μL of HEV stock (positive fecal samples identified in this study) was inoculated intravenously into each pig in the experimental group, and the three pigs in the other group served as negative controls. Serum and fecal samples were collected at 1 to 10 weeks post-inoculation (wpi) for alanine aminotransferase (ALT) levels, anti-HEV antibodies and HEV RNA detection, respectively. During necropsies, liver lesions and HEV antigen in liver were observed at 10 wpi.

**Results:**

The rate of fecal sample HEV RNA-positivity was 12% (12/100). Sequence comparisons indicated that partial ORF1 and ORF2 gene sequences of this isolate shared highest identities with corresponding sequences of genotype 4a HEV isolates (81.4%-96.1% and 89.9%-97.1%, respectively). Phylogenetic tree analysis further demonstrated that sequences of this isolate clustered together with sub-genotype 4a HEV isolate sequences. Experimentally, the pathogenicity of Bama miniature pigs infected with this isolate exhibited viremia, fecal virus shedding, seroconversion, ALT level increasing, liver lesions and HEV antigen in liver.

**Conclusions:**

This is the first study to confirm that HEV is currently circulating in laboratory Bama miniature pigs in China and this isolate can successfully infect Bama miniature pigs experimentally. More importantly, this study suggested HEV screening of laboratory pigs should be conducted to prevent research personnel from acquiring zoonotic HEV infections.

## Background

Hepatitis E virus (HEV) is a quasi-enveloped, single-stranded positive-sense RNA virus belonging to the family *Hepeviridae* [1]. This family contains two genera: *Orthohepevirus* (mainly mammalian hosts) and *Piscihepevirus* (cutthroat trout virus), with the former comprised of four species designated A-D [2]. The species *Orthohepevirus A* is classified into eight genotypes (HEV-1 through HEV-8) [3]. HEV-1 and HEV-2 are exclusively infectious to humans [4]. HEV-3 and HEV-4 are zoonotic (isolated from humans, swine, rabbits, cows, sheep, mongooses and deer) [5], while HEV-5 and HEV-6 mainly circulate in wild boars [6, 7] and HEV-7 and HEV-8 circulate in dromedary and Bactrian camels [8, 9], respectively. In China, the predominant HEV genotype detected in recent years, HEV-4, has been shown to spread via zoonotic transmission [10], and six subtypes of HEV-4 (4a, 4b, 4d, 4g, 4h and 4i) have been detected in humans and animals [10–13].

Lacking an efficient cell culture system, the process of studying HEV has been hampered in HEV propagation [14]. Researchers have had to use animals, such as non-human primates, swine, rabbits, mice and rats for most studies [15–17]. However, investigations based on rabbit, mouse and rat models have disadvantages when used to study clinical manifestations of HEV infection, while high costs, operational challenges and labor-intensive resource needs have limited the use of non-human primate and conventional swine models for such studies [18, 19]. Bama miniature pigs have served as a genetically stable, highly inbred, easily handled and low cost viable infection model that are currently used extensively in research, especially in long-term trials [20, 21]. Notably, recent studies have demonstrated that these animals are susceptible to experimental HEV infection and laboratory pigs harbor anti-HEV antibodies [14, 20], although HEV RNA detection in laboratory Bama miniature pig has not been reported to date. Based on these findings, this study was to investigate whether HEV is circulating in laboratory pigs.

## Results

### Amplification, comparisons and phylogenetic analysis of the partial ORF1 and ORF2 genes of HEV

The HEV RNA positivity rate was 12% (12/100) for the set of fecal samples that were collected from laboratory Bama miniature pigs. Firstly, a 280-bp region (primer binding sites were excluded) of the HEV ORF1 gene sequences were analyzed. The 12 sequences of this region shared 100% identity with each other and 67.5%-90.4% identity with other HEVs, with higher identity shared with isolates of known genotype 4 HEV strains and highest identity with genotype 4a sub-genotype isolates (86.1%-90.4%, Table 1). Meanwhile, similar results were obtained for the partial ORF2 gene: the 306-bp sequences shared 100% identity with each other and 76.1%-97.1% identity with other HEVs, with higher identity shared with known genotype 4 HEV isolates than other genotypes and highest identity shared with sub-genotype 4a isolates (89.9%-97.1%, Table 1).

**Table 1:** Comparisons of the CHN-SC-BMP1 (ORF1) and CHN-SC-BMP2 (ORF2) sequences obtained in the present study with the corresponding region of reported different HEV isolates in GenBank

HEV isolates	Accession No.	Identity (%)	
		CHN-SC-BMP1 (ORF1)	CHN-SC-BMP2 (ORF2)
Genotype 1	D10330, D11092, D11093	67.9-68.2	77.8-78.4
Genotype 2	KX578717	68.2	77.8
Genotype 3	AP003430, AY115488, FJ527832	67.5-70.4	76.1-77.8
Genotype 4a	AB197673, EF077630, EU366959, KC492825, MK410045	86.1-90.4	89.9-97.1
Genotype 4b	DQ279091, EU676172	81.8-83.2	84.3-85.3
Genotype 4c	AB074915, AB200239	80.0-82.1	84.6-86.3
Genotype 4d	AY594199, FJ610232, GU206559, GU361892, KF176351	78.6-80.4	83.7-85.3
Genotype 4e	AY723745	80.4	84.0
Genotype 4f	AB220974	80.4	85.3
Genotype 4g	AB108537	78.2	83.3
Genotype 4h	GU119961, GU188851	78.2-78.9	86.6-86.9
Genotype 4i	AB369690, DQ450072, HM439284	81.8-82.5	82.7-85.6

**Fig. 1:**
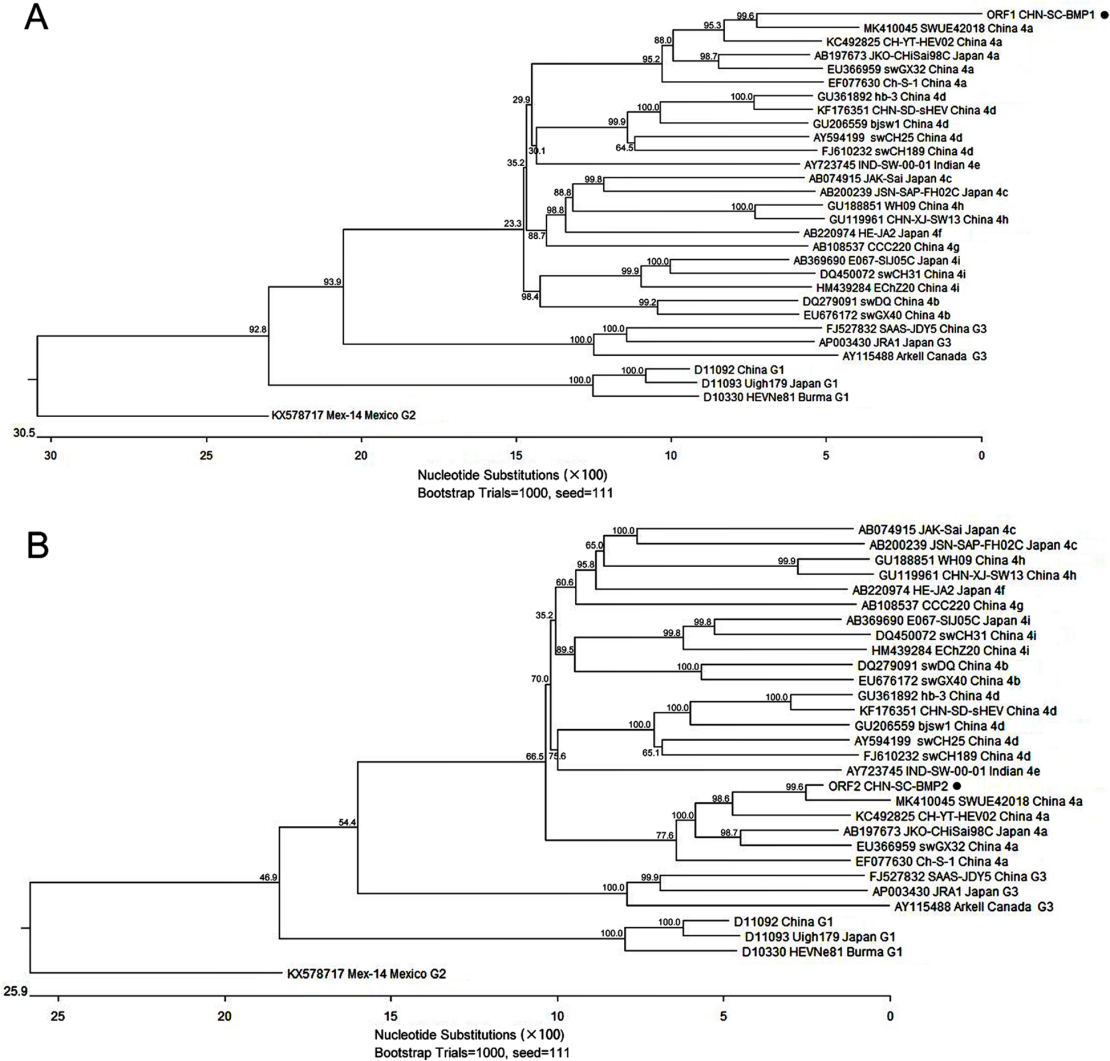
Phylogenetic tree based on the partial sequences of ORF1 (**A**) and ORF2 (**B**) obtained from Bama miniature pig feces. The phylogenetic tree was inferred by the neighbor-joining method (1,000 bootstrap replicates) using the MEGA 7 program. Sequences of CHN-SC-BMP1 (ORF1) and CHN-SC-BMP2 (ORF2) are labeled with “●”

Further phylogenetic tree analysis of 30 genomes of HEV1-HEV4 strains and the strain detected in this study confirmed this isolate belonged to genotype 4 HEV (Fig. 1). Meanwhile, according to the sequences of CHN-SC-BMP1 (ORF1) and CHN-SC-BMP2 (ORF2), this isolate was also confirmed to cluster with sub-genotype 4a HEV isolates (Fig. 1).

### Seroconversion, viremia, fecal virus shedding, and ALT levels in the experimentally infected pigs

**Fig. 2:**
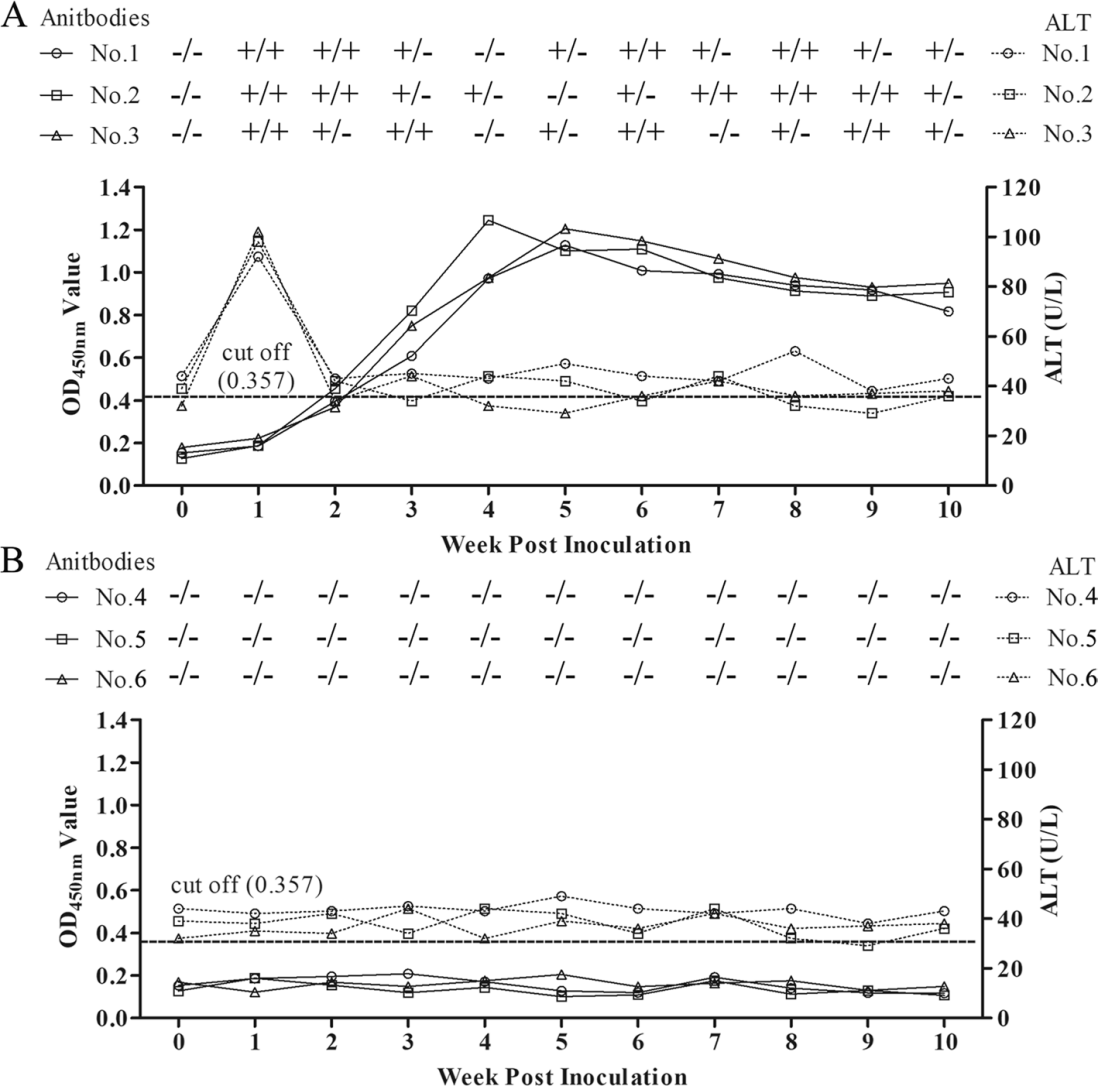
Fecal viral shedding / viremia, ALT levels, and antibody levels in pigs experimentally inoculated with the virus isolated in this study. (**A**) Inoculated group; (**B**) Negative control group. “+” and “-” represent positive and negative for fecal virus shedding and viremia, respectively. Detection of swine HEV RNA using RT-nPCR.

In the inoculated group, all pigs seroconverted (cut-off value was 0.357) at 2 week post inoculation (wpi) and then reached a peak level (4-5 wpi) (Fig. 2A), and at the ends of the experiment, pigs were still positive (10 wpi) (Fig. 2A). In contrast, all control pigs were seronegative throughout the study (Fig. 2B). The fecal and serum samples from all pigs were negative for swine HEV RNA at pre-inoculation, and pigs remained negative throughout the experiment in the negative control group (Fig. 2B). Fecal virus shedding and viremia were first detected in all inoculated pigs at 1 wpi (Fig. 2A). Meanwhile, HEV RNA was detectable in fecal samples until 10 wpi in all pigs and was detected in serum samples for 7-8 weeks (Fig. 2A).

During the entire study, there was no elevation of ALT levels in serum samples in negative control group (Fig. 2B). While ALT levels transiently increased (92-102 U/L) at 1 wpi and then returned to baseline levels in all inoculated pigs (Fig. 2A). Meanwhile, the increasing ALT levels from inoculated pigs were higher than the ones in negative control group at 1 wpi, but not at other wpi.

### Histopathological and immunohistochemical changes in liver tissues

**Fig. 3:**
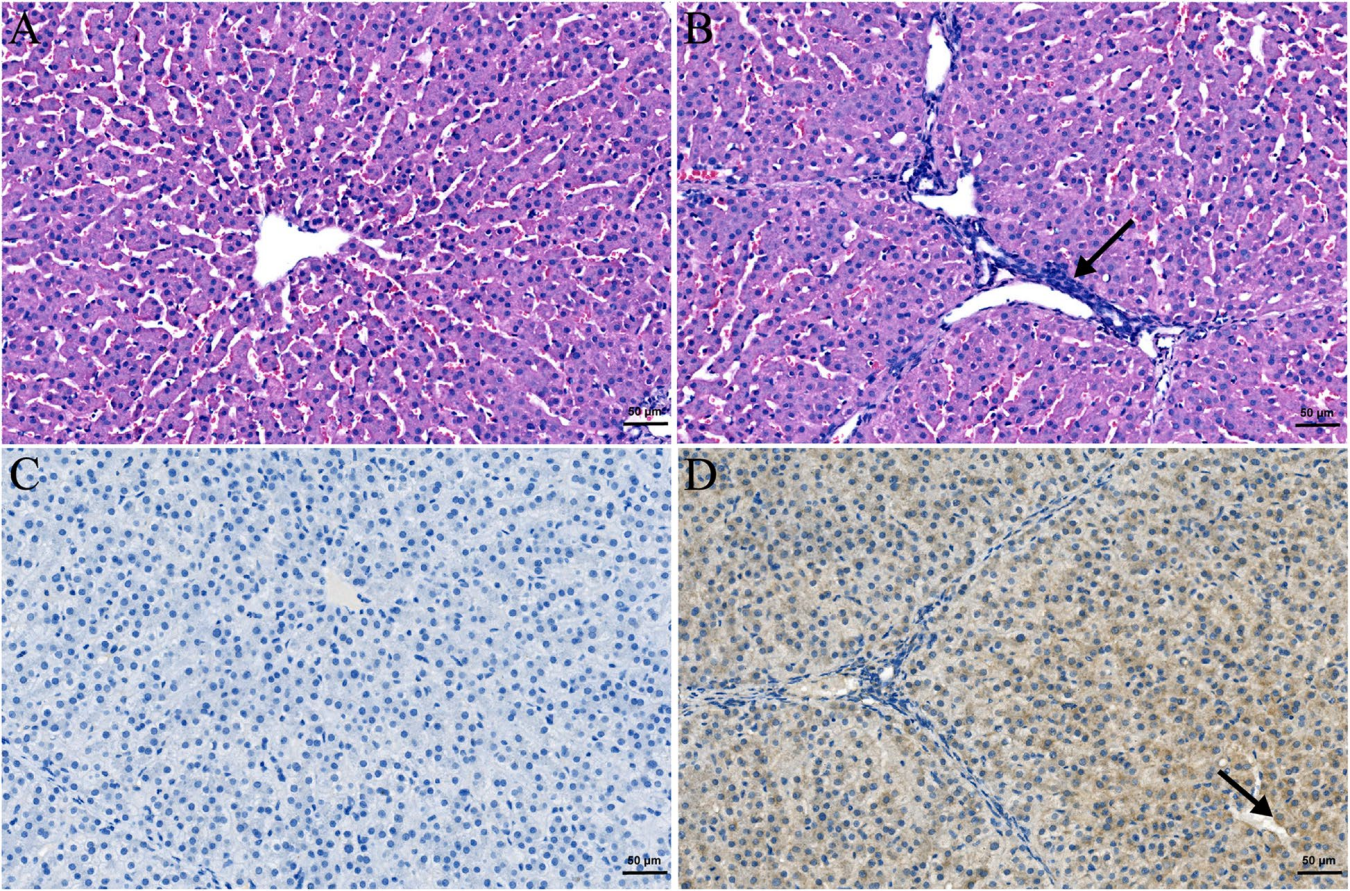
Microscopic lesions and HEV antigens detection in the liver from the necropsied pigs. (**A**) Liver sections from pigs in negative control group showing no visible pathological signs of HEV infection; (**B**) Local lymphocytic venous periphlebitis (arrow) in inoculated pigs; (**C**) Liver sections from pigs showing no specific brown staining in negative control group; (**D**) HEV antigens in the livers (arrow) of all inoculated pigs

Microscopically, all pigs in the negative control group had no hepatic lesions (Fig. 3A), while all inoculated pigs showed local lymphocytic portal phlebitis in the livers (Fig. 3B). With IHC staining, no specific brown staining was observed in the livers of all uninoculated pigs (Fig. 3C), and HEV antigens were detected in the inoculated group (Fig. 3D).

## Discussion

At present, non-human primates, including swine, rabbits, mice and rats, usually served as experimental subjects in most HEV studies [15–17], but all these animal models have shortcomings [18, 19] . By contrast, Bama miniature pigs are an ideal infection model used extensively in research, especially for long-term trials [20, 21]. In previous studies, antibodies specific for HEV have been detected in laboratory Bama miniature pigs, an animal host also known to be susceptible to experimental HEV infection [14, 20]. The results in this study showed that HEV RNA positivity rate was 12% (12/100) from collected fecal samples of laboratory Bama miniature pigs in Sichuan Province, and the partial ORF1 and ORF2 gene sequences of this isolate shared highest identities with corresponding sequences of genotype 4a HEV isolates, respectively. The pathogenicity of this isolate in Bama miniature pigs was characterized by emerged viremia, fecal virus shedding, seroconversion, ALT level increasing, liver lesions and HEV antigen in liver. This is the first study to detect HEV RNA in fecal samples from laboratory Bama miniature pigs, and this HEV strain can successfully infect Bama miniature pigs experimentally.

In China, HEV-4 has been shown the predominant circulating genotype in recent years, and six subtypes of HEV-4 (4a, 4b, 4d, 4g, 4h and 4i) have been detected in humans and animals [10–13]. Interestingly, research had shown that HEV-4 subtype distribution varied among different region in China: HEV-4a was the predominant subtype in humans and pigs in eastern China, while HEV-4b was in southern China [22]. In the present study, this HEV strain from laboratory Bama miniature pigs in southwestern China belonged to HEV-4a genotype. Importantly, this finding suggests that HEV-4a may circulate currently in laboratory Bama miniature pigs in this region, and more epidemiological research needs to be done to confirm this hypothesis in future studies.

In the experimental inoculation study, all inoculated pigs exhibited viremia, fecal virus shedding, seroconversion, increasing ALT level, liver lesions and HEV antigen in liver, and the pathogenicity was similar as swine HEV inoculated Bama miniature pigs as previously described [20]. However, there were obvious differences between these two findings for the time of viremia, fecal virus shedding and seroconversion, the experimental pathogenicity study showed that the virus in pigs lasted until 10 weeks, which was longer than previous study [20]. The main reason for this difference was the virus doses dependent as well as the various genotypes of swine HEV. Meanwhile, there is an oscillation of the RNA detection in blood and stools in inoculated group, which have been also found in previous studies [17, 23]. The possible reason is that the sensitivity of the RT-nPCR assays affected the results. It is well-known that swine HEV-4 is zoonotic and can infect cynomolgus macaques, rabbits, BALB/c mice and humans [17, 22, 24, 25]. Therefore, HEV screening of laboratory Bama miniature pigs should be conducted as a precautionary measure to reduce risk of zoonotic HEV transmission from pigs to laboratory personnel in the work environment.

## Conclusion

Collectively, our results show that HEV is currently circulating in laboratory Bama miniature pigs in China and this isolate can successfully infect Bama miniature pigs experimentally. More importantly, our findings emphasize that HEV screening of laboratory Bama miniature pigs should be performed to ensure the usefulness of this model for studying clinical manifestations of HEV, and also preventing zoonotic HEV transmission from pigs to research personnel.

## Methods

### Clinical sample collection and processing

In December 2020, one hundred rectal swabs were collected from Bama miniature pigs at a laboratory animal center in Sichuan Province, southwest China. Each sample was diluted in phosphate-buffered saline to generate a 10% (w/v) fecal homogenate suspension, followed by clarification of suspensions via centrifugation at 4500 × g for 10 min at 4 °C.

### Amplification of the partial ORF1 and ORF2 genes of HEV

200 μL of 10 % fecal suspension was used for total RNA extraction by TRIzol Reagent (TaKaRa, China). All samples were analyzed using a broad-spectrum nested reverse transcription polymerase chain reaction (RT-nPCR) with specific primers designed to amplify the partial RdRp region of HEV ORF1 gene, which were described previously by Reimar Johne [26]. In addition, to confirm detection of HEV, the partial ORF2 gene of the HEV genome was also amplified using RT-nPCR as described previously [27]. Briefly, for RT-nPCR, reverse transcription and first PCR were performed using PrimeScript™ One Step RT-PCR Kit (TaKaRa, China). Next, the second PCR was conducted using TransTaq High Fidelity DNA polymerase (TransGen Biotech, China) based on the manufacturer’s instructions. Finally, PCR products were identified by electrophoresis on 1% agarose gel. To avoid cross-contamination, the negative controls were set up in all of these experiments, and filter tips were also used throughout the process. All purified positive PCR products were sequenced by Genetic Analyzer(ABI 3130, Applied Biosystems, USA). Two sequences were submitted to GenBank (Accession numbers MW498242 and MW498243).

### Sequence analysis

Based on sequences that were obtained, multiple alignments were performed using the MegAlign program within the Lasergene software package (Version:7.1.0, DNASTAR Inc., Madison, WI). Next, phylogenetic trees were constructed that also incorporated other known GenBank HEV strain sequences using the MEGA7 software. GenBank numbers included D10330, D11092, D11093, KX578717, FJ527832, AY115488, AP003430, AB197673, EF077630, EU366959, KC492825, MK410045, DQ279091, EU676172, AB074915, AB200239, AY594199, FJ610232, GU206559, GU361892, KF176351, AY723745, AB220974, AB108537, GU119961, GU188851, AB369690, DQ450072 and HM439284.

### Animal experiment design and samples collection

Six healthy Bama miniature pigs (body weight, 5 kg) were randomly divided into two groups of 3 pigs each, and were monitored for 2 weeks to ensure negative HEV RNA and antibody test before challenged. The virus (a pool of HEV-4a from faeces of all HEV positive Bama pigs analyzed earlier) was quantitatively analyzed by RT-nPCR as previously described [28] and the titer of this infectious stock was 10^6^ genome equivalents per ml (10^6^ GE/ml). A total of 500 μL of this stock was inoculated intravenously into each pig in the experimental group, and the three pigs in the other group served as negative controls. Fecal and serum samples were collected from each pig before inoculation and weekly thereafter. Serum samples were tested for alanine aminotransferase (ALT) levels and anti-HEV antibodies. Fecal and serum samples were also tested for HEVs RNA by RT-nPCR. After pigs were necropsied at 10 wpi, liver samples were collected and fixed in 10% neutral buffered formalin for histological examination and immunohistochemistry (IHC).

### Detection of anti-HEV antibodies , ALT concentrations and swine HEV RNA

Anti-swine HEV IgG antibodies were tested in serum samples by indirect ELISA as previously described [12]. Briefly, purified CHN-SD-sHEV truncated capsid protein (200 ng/well) was coated on the plates overnight at 4°C. After blocked and washed, serum samples (1:100, 100 μL/well) were added into each well and incubated for 1 hour at room temperature (RT). After washed, horseradish peroxidase (HRP)-conjugated goat anti-swine IgG (Jackson ImmunoResearch, West Grove, PA, USA) (1:5000, 100 μL/well) was added and also incubated for 1 hour. After washed again, 3,3´,5,5´-tetramethylbenzidine (TMB) was added and the plates were incubated in the dark for 15 min at RT. The colorimetric reaction was stopped (3 M H_2_SO_4_, 50 μL/well) and optical density (OD) values were read at 450 nm by an automated microplate reader (Bio-Rad, USA). Each sample was detected in duplicate wells.

ALT concentrations in plasma samples from pigs were measured using standard methods on a SmartSpec 3000 spectrophotometer. Before challenged, the serum sample was collected weekly for three times from each pig and the average ALT baseline was 38 U/L (physiological ALT range: 32-44 U/L). Pigs were considered positive for hepatitis, when their ALT levels exceeded pre-challenge ALT levels more than two-fold [29].

The partial ORF2 gene of swine HEV RNA was amplified from 200 μL 10 % fecal suspension or 200 μL sera from inoculated pigs were tested according to the same method as described above.

### Evaluation of histopathological and immunohistochemical changes in liver tissues

During necropsies, the liver tissues were harvested separately and fixed for routine histological examination. IHC analyses were conducted using an Ultrasensitive^TM^ SP kit and a DAB Detection Kit (Fuzhou Maixin Biotechnology Development Co., China) based on the manufacturer’s instructions. The monoclonal antibody 3E8 (mouse anti-HEV capsid protein, 1mg/ml, 1:1,000 dilution) was used.

## Data Availability

The datasets generated and/or analysed during the current study are available in the [NCBI] repository, [ACCESSION NUMBERS MW498242 and MW498243]”.

## Abbreviations

HEV: Hepatitis E virus
ELISA: Enzyme-linked immunosorbent assay
ORF: Open Reading Frame
RT-nPCR: Reverse Transcription-nested Polymerase Chain Reaction
wpi: Week post inoculation
IHC: Immunohistochemistry
